# Fused Thiopyrano[2,3-*d*]thiazole Derivatives as Potential Anticancer Agents

**DOI:** 10.3797/scipharm.1204-02

**Published:** 2012-05-03

**Authors:** Anna Kryshchyshyn, Dmytro Atamanyuk, Roman Lesyk

**Affiliations:** 1 Department of Pharmaceutical, Organic and Bioorganic Chemistry, Danylo Halytsky Lviv National Medical University, Pekarska 69, 79010, Lviv, Ukraine.; 2 Mutabilis, 102 Avenue Gaston Roussel, 93230 Romainville, France.

**Keywords:** Thiopyrano[2,3-*d*][1,3]thiazoles, Alkylation, Cyanoethylation, Anticancer activity, COMPARE analysis

## Abstract

*rel*-(5a*R*,11b*R*)-3,5a,6,11b-tetrahydro-2*H*,5*H*-chromeno[4′,3′:4,5]thiopyrano[2,3-*d*][1,3]thiazol-2-ones formed by the stereoselective Knoevenagel-*hetero*-Diels-Alder reaction were functionalized at the nitrogen in position 3 via reactions of alkylation, cyanoethylation, and acylation. The synthesized compounds were evaluated for their anticancer activity in NCI60 cell lines. Among the tested compounds, **3f** was found to be the most active candidate with the greatest influence on leukemia, non-small cell lung cancer, colon cancer, CNS cancer, melanoma, prostate cancer, and breast cancer subpanel cell lines with GI_50_ values over a range of 0.37–0.67 μM.

## Introduction

Thiopyrano[2,3-*d*]thiazole derivatives as well as their synthetic precursors, namely 5-ylidene-4-thiazolidinones [1, 2], exhibit various pharmacological activities and may be considered as a possible source of innovative drug-candidates. The earliest reports of thiopyrano[2,3-*d*]thiazole biological activity is related to the antifungal and antituberculosis activities. It is important to note that these derivatives were highly active towards pathogenic isolates of *Trichoderma harzianum*, *Penicillium simplex*, *Candida albicans*, *Mucor hiemalis*, *Aspergillus oryzae*, *Actynomices sp.*, *A. fumigatus* at the level of fluconazole [3–5].

**Fig. 1:**
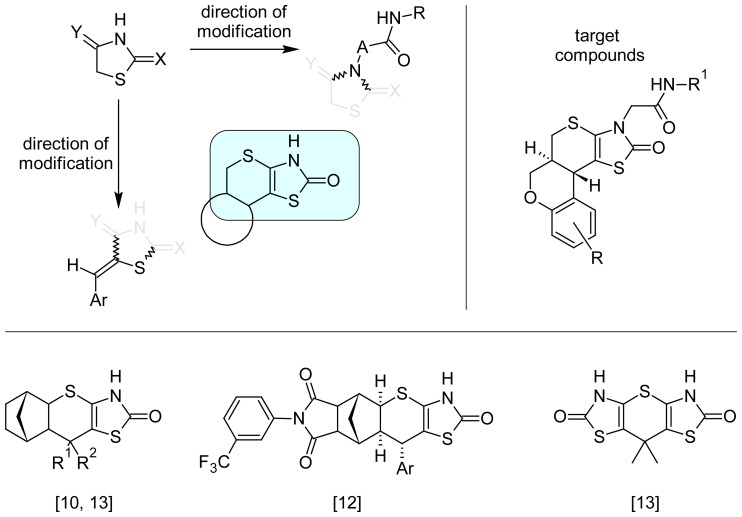
Background for the synthesis of the target compounds

Modern research allowed the identification of antitumor potential of different 4-thiazolidin-one derivatives and abovementioned heterocycles [6]. Moreover, some directions of highly active compounds design and optimization have been proposed (figure 1). Approach to the design of target compounds was based on the number of generated hypotheses and facts. The presence of substitutes at the C5 position of basic heterocycle is critical for 4-thiazolidinone derivatives antitumor effect realization and value [1, 2, 7–9]. Fixation of highly active 5-arylidene-4-thiazolidinone in thiopyranothiazole system usually saves the activity vector and opens up new possibilities of obtained derivatives optimization [10–13]. Moreover, the introduction of different substitutes in the N3 position is one of the effective methods of target compounds optimization. This approach allowed substantive increasing in the level and/or selectivity of the investigated compounds antitumor effect in comparison with N-unsubstituted analogues [6, 8, 14].

## Results and Discussion

### Chemistry

Tetracyclic starting compounds (5aR*,11bR*)-3,5a,6,11b-tetrahydro-2*H*,5*H*-chromeno-[4′,3′:4,5]thiopyrano[2,3-*d*]thiazoles **1a–c** were obtained in the Knoevenagel-*hetero*-Diels-Alder reaction via isorhodanine (4-thioxo-2-thiazolidinone) and appropriate 2-allyloxy-benzaldehydes interaction in acetic medium at room temperature (scheme 1). According to the ^1^H NMR of the **1a–c** trans annulated cycloadducts were formed. The configuration of **1a–c** was deduced from the coupling constant (J = 10.5 Hz) between the 5a and 11b protons [11].

Starting **1a**–**c** contain NH-acidic centers at position N3 of basic heterocycles that from chemical point of view was the rationale for the synthesis of various 3-substituted derivatives. Such synthetic approach is reasonable because of anticancer activity intensification of structurally simpler N-3-substituted 4-azolidinones containing carboxylic acids moieties in 3-d position. On the other hand SAR analysis showed that simultaneous presence of substitutes in the positions C5 and N3 of basic heterocycle is perspective direction of new antitumor agents rational design based on 4-azolidinones and related heterocycles [1, 6, 8].

Alkylation reactions of **1a–c** were carried out through the stage of potassium salts formation that later were used in the reactions with bromoacetophenones, ethylchloro-acetate and different acetamides (Scheme 2).

To expand the number of N-substituted chromeno[4′,3′:4,5]thiopyrano[2,3-*d*]thiazole-2-one derivatives and obtain new pharmacologically attractive structures 3-(2-carboxyethyl)-chromeno[4′,3′:4,5]thiopyrano[2,3-*d*]thiazole-2-one **5** was synthesized (scheme 3). We worked out two alternative methods of its synthesis. The first one is based on the alkylation reaction of starting compound with sodium 3-chloropropionate via intermediate potassium salt. The above method has a significant disadvantage. The target product contains impurities of starting substance and requires multistage purification. In technological term the best is the second method we have proposed, based on the cyanoethylation reaction. So, via interaction of the compound **1a** with acrylonitrile in the pyridine medium propionitrile **4** was obtained with high yield. Hydrolysis of the latter allowed obtainment of target acid **5** qualitative characteristics of which far outweigh the product obtained in the alkylation reaction. For carboxylic group functionalization corresponding acid chloride was synthesized and used in the reaction of aromatic amines acylation.

### Biological activity

The main focus of biological activity studies was the search for compounds with antitumor activity. The newly synthesized compounds were selected by the National Cancer Institute (NCI) within the Developmental Therapeutic Program (www.dtp.nci.nih.gov) for *in vitro* cell line screening. Anticancer assays were performed according to US NCI protocol, which was described elsewhere [15–18]. The compounds were first evaluated at one dose primary anticancer assay towards approximately 60 cell lines (concentration 10^−5^ M). The human tumor cell lines represent all forms of cancer (such as non-small cell lung cancer, colon cancer, breast cancer, ovarian cancer, leukemia, renal cancer, melanoma, prostate cancer). In the screening protocol, each cell line was inoculated and pre-incubated for 24–48 h on a microtiter plate. Test agents were then added at single concentration and the culture was incubated for an additional 48 h. The end point determinations were made with a protein binding dye, sulforhodamine B (SRB). The results for each test agent were reported as the percent growth of the treated cells compared to the untreated control cells. The preliminary screening results are shown in table 1.

Among tested chromeno[4′,3′:4,5]thiopyrano[2,3-*d*]thiazoles except highly active **3a** and **3b** selected for the in-depth study, noteworthy is the 3-substituted propionitrile **4**, for which there was observed the selective effect on breast cancer line HS 578T (GP = 8.59%). Other compounds tested in one-dose primary assay didn’t show any impressive anticancer activity and therefore can’t be considered as prospective anticancer agents.

Compounds **3a**, **3b**, **3f**, **3h**, **3i**, **3n**, **3p–r** were tested on 60 tumor cell lines over a range of concentrations (10^−4^–10^−8^). In this assay three dose-responce parameters are obtained:

1. growth inhibition of 50% – **GI_50_**;
2. total growth inhibition – **TGI**;
3. **LC_50_**.

Whereas the **GI_50_** may be viewed as a growth-inhibitory level of effect, the **TGI** signifies a “total growth inhibition” or cytostatic level of effect. The **LC_50_** is the lethal concentration, “net cell killing” or cytotoxity parameter. If tested parameters (logGI_50_, logTGI and logLC_50_) specified in negative log units are less then < −4.00 these compounds are assigned as active [19–21]. Results estimation of the compound **5** showed that the most sensitive tumor cell lines to this compound are leukemia.

Analyzing the results of a detailed *in vitro* screening tested chromeno[4′,3′:4,5]–thiopyrano[2,3-*d*]thiazoles except **3n** and **3p** showed significant growth inhibition; the average value of logGI_50_ ranges from −4.49 to −6.22 (table 2). The highest anticancer effect was observed for compounds **3a**, **3f** and **3q** that showed significant cytostatic effect on almost all tumor cell lines. Thus for the obtained compounds there was found no expressed selectivity of action toward separate cancer lines or cancer types. The only exception is the compound **3n**, which against moderate activity (average value loogGI_50_ is −4.14) caused inhibition of cell growth of leukemia lines *CCRF-CEM* and *HL-60(TB)* in submicromolar concentrations (logGI_50_ – −6.93 and < −8.00, respectively).

Functionalization of the position N3 of basic heterocycle structure by introduction of acetamide molecular fragments is the most effective direction for anticancer profile optimization of chromeno[4′,3′:4,5]thiopyrano[2,3-*d*]thiazoles. Results of our work may be used for planning the directed synthesis of potential anticancer agents.

Taking into consideration high antitumor potential of the compounds **3a, 3f** and **3q** and prospects for their enhanced pharmacological studies we have calculated the quantitative indicators of selectivity effects on different types of cancer based on the experimental results of in-depth screening. The selectivity ratios (SR) were calculated by dividing the full panel MID GI_50_ and MID TGI (the average sensitivity of all cell lines towards the test agent) by their individual subpanel MID (the average sensitivity of all cell lines of a particular subpanel towards the test agent). Selectivity ratios of 3–6 refer to moderate selectivity; ratios greater than 6 indicate high selectivity towards the corresponding cell line, while compounds not meeting either of these criteria are rated non-selective.

Compound **3a** was found to be non-selective (SR were between 0.66–2.06 and 0.84–1.66 at the GI_50_ and TGI levels respectively). Two other “hit-compounds” **3f** and **3q** showed moderate selectivity toward the prostate cancer subpanel with selectivity ratios SR = 3.24 at GI_50_ level for **3f** and SR = 4.41 at TGI level for **3q**.

**COMPARE analysis** was performed for compounds **3a**, **3b** and **3f** to investigate the similarity of their cytotoxicity pattern (mean graph fingerprints) with those of known anticancer standard agents, NCI active synthetic compounds and natural extracts, which are present in public available databases (http://dtp.nci.nih.gov/docs/compare/......compare.html) [22–25]. Such analysis is based on comparing the patterns of differential growth inhibition for cultured cell lines and can potentially gain insight into the mechanism of the cytotoxic action. If the data pattern correlates well with that of compounds belonging to a standard agent database (Pearson’s correlation coefficient (PCC) >0.6), the compound of interest may have the same mechanism of action. On the other hand, if the activity pattern does not correlate with any standard agent, it is possible that the compound has a novel mechanism of action. Standard COMPARE analysis was performed at the GI_50_ level.

It was established that tested compounds do not have any high correlation levels with the NCI tested drugs or other biological active substances. This may be an argument in favor of the compounds unique mechanism of action that differs from the traditional links of influence on oncogenesis of known anticancer agents. However in our opinion some COMPARE analysis results deserve attention. Thus, there is correlation of the “hit-compounds” anticancer profile with macbecin II (NSC:S330500 Endpt:GI50 ExpId:AVGDATA hiConc: −6.0) which is DNA antimetabolite, which mechanism of action is in part due to heat shock protein Hsp90 protein inhibition [26]. Also one should mention that when comparing the GI_50_ values on each cancer cell line for 3f there is some correlation with rhizoxin as inhibitor of tubulin (NSC:S332598 Endpt:GI50 ExpId:AVGDATA hiConc: −5.3) p=0.686. It is interesting that among the possible ways of “hit-compounds” antitumor effect realization COMPARE analysis does not deny the possible induction of apoptosis, which is an argument for the experimental confirmation of this hypothesis considering the anticancer mechanism of action of structurally related 5-(4-methylbenzylidene)-2-phenylamino-1,3-thiazolidin-4-one (MMPT) [27].

## Experimental

### Chemistry

Melting points were measured in open capillary tubes on a BUCHI B-545 melting point apparatus and are uncorrected. The elemental analyses (C, H, N) were performed using the Perkin–Elmer 2400 CHN analyzer and were within 0.4% of the theoretical values. The ^1^H- and ^13^C-NMR spectra were recorded on Varian Gemini 400 MHz or Bruker 125 MHz for frequencies 100 MHz in DMSO-*d*_6_ using tetramethylsilane as an internal standard. Chemical shifts are reported in ppm units with use of d scale. Mass spectra were obtained using electrospray (ES) ionization techniques on an Agilent 1100 Series LCMS.

### Preparation of compounds 1a–c

A mixture of appropriate 2-allyloxybenzaldehyde (0.01 mol), 4-thioxo-2-thiazolidinone (0.01 mol) and acetic acid or acetonitryle (20 ml) was stirred at room temperature in the presence of triethylamine (0.01 mol). Obtained solid product was collected by filtration and recrystallized from dioxane.

#### rel-(5aR,11bR)-3,5a,6,11b-Tetrahydro-2H,5H-chromeno[4′,3′:4,5]thiopyrano[2,3-d]–[1,3]thiazol-2-one (**1a**)

Yield: 80%, mp 230–231°C (Dioxane) [11]. ^1^H NMR (400 MHz, DMSO-*d_6_*): 2.22–2.36 (m, 1H, 5a-H), 3.00–3.08 (m, 1H, 5-H), 3.15 (dd, 1H, *J* = 3.6, 12.0 Hz, 5-H), 3.82–4.00 (m, 1H, 6-H), 3.97 (d, 1H, *J* = 10.5 Hz, 11b-H), 4.39 (dd, 1H, *J* = 3.6, 10.3 Hz, 6-H), 6.85 (d, 1H, J = 7.6 Hz, 8-H), 6.95 (t, 1H, *J* = 7.8 Hz, 10-H), 7.17 (t, 1H, *J* = 7.6 Hz, 9-H), 7.43 (d, 1H, *J* = 7.8 Hz, 11-H), 11.50 (s, 1H, NH). ^13^C NMR (100 MHz, DMSO-*d*_6_): 27.9, 30.2, 38.7, 69.2, 105.6, 117.4, 121.2, 121.6, 123.4, 127.7, 129.2, 153.7, 170.9. ESI-MS *m/z* 278 (M+H)^+^. Anal. Calcd for C_13_H_11_NO_2_S_2_, %: C, 56.30; H, 4.00; N, 5.05. Found, % C, 56.35; H,4.06; N, 5.04.

#### rel-(5aR,11bR)-10-Bromo-3,5a,6,11b-tetrahydro-2H,5H-chromeno[4′,3′:4,5]thiopyrano–[2,3-d][1,3]thiazol-2-one (**1b**)

Yield: 83%, mp 237–239°C (AcOH). ^1^H NMR (400 MHz, DMSO-*d_6_*): 2.45 (m, 1H, 5a-H), 2.95–3.10 (m, 2H, 2*5-H), 3.90–4.00 (m, 1H, 6-H), 4.08 (d, 1H, *J* = 10.5 Hz, 11b-H), 4.40 (dd, 1H, *J* = 3.8, 10.8 Hz, 6-H), 6.78 (d, 1H, *J* = 8.0 Hz, 8-H), 7.10 (d, 1H, J = 8.0 Hz, 9-H), 7.44 (s, 1H, 11-H), 11.35 (s, 1H, NH). ESI-MS *m/z* 356 and 358 (M+H)^+^. Anal. Calcd for C_13_H_10_BrNO_2_S_2_, %: C, 43.83; H, 2.83; N, 3.93. Found, %: C, 44.00; H, 2.79; N, 3.93.

#### rel-(5aR,11bR)-10-Nitro-3,5a,6,11b-tetrahydro-2H,5H-chromeno[4′,3′:4,5]thiopyrano– [2,3-d][1,3]thiazol-2-one (**1c**)

Yield: 55%, mp 214–216°C (AcOH). ^1^H NMR (400 MHz, DMSO-*d_6_*): 2.42 (m, 1H, 5a-H), 3.09–3.16 (m, 1H, 5-H), 3.45 (dd, 1H, *J* = 3.6, 12.0 Hz, 5-H), 4.00–4.11 (m, 1H, 6-H), 4.22 (d, 1H, *J* = 10.5 Hz, 11b-H), 4.48 (dd, 1H, *J* = 3.6, 10.3 Hz, 6-H), 7.06 (d, 1H, *J* = 7.6 Hz, 8-H), 8.10 (d, 1H, *J* = 7.6 Hz, 9-H), 8.21 (s, 1H, 11-H), 11.50 (s, 1H, NH). ESI-MS *m/z* 323 (M+H)^+^. Anal. Calcd for C_13_H_10_N_2_O_4_S_2_, %: C, 48.44; H, 3.13; N, 8.69. Found, %: C, 48.64; H, 3.25; N, 8.80.

### Preparation of for potassium salts (2a–c)

To a stirred suspension of **1a–c** (0.01 mol) in 30 ml of ethanol potassium hydroxide (0.011 mol) in 15 ml of ethanol was added. Reaction mixture was stirred at room temperature for 1 h. The formed potassium salt was filtered, washed with ethanol, diethyl ether and used in the following transformations without additional purification.

### Preparation of compounds 3c,d

To a suspension of **2a** (0.01mol) in the ethanol 0.011 mol of *p*-fluoro- or *p*-chloro-bromoacetophenone was added. The reaction mixtures were refluxed for 3–9 h, the progress of the reactions was monitored by TLC; formed precipitates were collected by filtration and crystallized from appropriate solvents.

#### rel-(5aR,11bR)-3-[2-(4-Fluorophenyl)-2-oxoethyl]-3,5a,6,11b-tetrahydro-2H,5H-chromeno[4′,3′:4,5]thiopyrano[2,3-d][1,3]thiazol-2-one (**3c**)

Yield 78%, mp 178–180°C (MeOH). ^1^H NMR (400 MHz, DMSO-*d_6_*): 2.10–2.35 (m, 1H, 5a-H), 3.00 (t, 1H, *J* = 11.6 Hz, 5-H), 3.15 (dd, 1H, *J* = 4.1, 12.0 Hz, 5-H), 3.90 (t, 1H, *J* = 10.4 Hz, 6-H), 4.10 (d, 1H, *J* = 10.4 Hz, 11b-H), 4.40 (dd, 1H, *J* = 3.8, 10.4 Hz, 6-H), 5.25 (s, 2H, CH_2_CO), 6.85 (d, 1H, *J* = 8.1 Hz, 8-H), 7.00 (t, 1H, *J* = 7.8 Hz, 10-H), 7.21 (t, 1H, *J* = 7.6 Hz, 9-H), 7.40 (d, 1H, *J* = 8.1 Hz, 11-H), 7.50 (m, 2H, 4-F-C_6_H_4_), 8.15 (dd, 2H, *J* = 5.5, 8.2 Hz, 4-F-C_6_H_4_). ^13^C NMR (100 MHz, DMSO-*d*_6_): 28.4, 38.0, 39.0, 50.0, 68.7, 105.6, 116.6 (d, *J*_CF_ = 22 Hz, 2C), 117.7, 121.2, 122.9, 124.7, 127.7, 128.9, 131.3, 131.8 (d, *J*_CF_ = 10 Hz, 2C), 154.9, 166.1 (d, *J*_CF_ = 202 Hz, 1C), 169.7, 191.3. ESI-MS *m/z* 415 (M+H)^+^. Anal. Calcd for C_21_H_16_FNO_3_S_2_, %: C, 61.00; H, 3.90; N, 3.39. Found, %: C, 61.18; H, 3.75; N, 3.47.

#### rel-(5aR,11bR)-3-[2-(4-Chlorophenyl)-2-oxoethyl]-3,5a,6,11b-tetrahydro-2H,5H-chromeno[4′,3′:4,5]thiopyrano[2,3-d][1,3]thiazol-2-one (**3d**)

Yield 87%, mp 160–162°C (acetonotrile). ^1^H NMR (400 MHz, DMSO-*d_6_*): 2.10–2.30 (m, 1H, 5a-H), 3.00 (t, 1H, *J* = 10.6 Hz, 5-H), 3.18 (dd, 1H, *J* = 4.0, 11.3 Hz, 5-H), 3.88 (t, 1H, *J* = 11.1 Hz, 6-H), 4.10 (d, 1H, *J* = 10.5 Hz, 11b-H), 4.38 (dd, 1H, *J* = 3.7, 10.4 Hz, 6-H), 5.25 (s, 2H, CH_2_CO), 6.84 (d, 1H, *J* = 8,1 Hz, 8-H), 6.97 (t, 1H, *J* = 8.2 Hz, 10-H), 7.22 (t, 1H, *J* = 7.6 Hz, 9-H), 7.45 (d, 1H, *J* = 7.7 Hz, 11-H), 7.60 (d, 2H, *J* = 8.8 Hz, 4-Cl-C_6_H_4_), 8.15 (d, 2H, *J* = 8.8 Hz, 4-Cl-C_6_H_4_). ^13^C NMR (100 MHz, DMSO-*d*_6_): 27.9, 33.8, 38.0, 49.8, 66.2, 105.2, 117.3, 121.0, 123.2, 127.6, 129.2, 129.6, 130.3, 130.6, 133.1, 139.8, 153.6, 170.1, 191.8. ESI-MS *m/z* 431 and 433 (M+H)^+^. Anal. Calcd for C_21_H_16_ClNO_3_S_2_, %: C, 58.67; H, 3.75; N, 3.26. Found, %: C, 58.84; H, 3.63; N, 3.29.

### Preparation of 3a,b and 3e–t

The mixture of 0.01 mol **2a–c**, 0,015 mol of ethylchloroacetate or appropriate chloroacetamide, 5 ml of DMF, 15 ml of ethanol and catalytic amounts of potassium iodide and potassium carbonate was refluxed for 3 h. The product of the reaction was collected by filtration, washed with water and diethyl ether. Esters **3a,b** were recrystallized from ethanol. Amides **3e–t** were recrystallized from a mixture of DMF–ethanol (1:2)

#### Ethyl rel-[(5aR,11bR)-2-oxo-5a,11b-dihydro-2H,5H-chromeno[4′,3′:4,5]thiopyrano–[2,3-d][1,3]thiazol-3(6H)-yl]acetate (**3a**)

Yield 68%, mp 132–134°C (EtOH). ^1^H NMR (400 MHz, DMSO-*d_6_*): 1.20 (t, 3H, CH_3_), 2.05–2.30 (m, 1H, 5a-H), 3.03 (t, 1H, *J* = 11.8 Hz, 5-H), 3.22 (dd, 1H, *J* = 4.0, 11.8 Hz, 5-H), 3.88 (t, 1H, *J* = 10.7 Hz, 6-H), 4.01 (d, 1H, *J* = 10.5 Hz, 11b-H), 4.16 (q, 2H, OCH_2_), 4.41 (dd, 1H, *J* = 3.9, 10.7 Hz, 6-H), 4.48 (s, 2H, CH_2_CO), 6.88 (d, 1H, *J* = 8.1 Hz, 8-H), 6.96 (t, 1H, *J* = 7.5 Hz, 10-H), 7.19 (t, 1H, *J* = 8.1 Hz, 9-H), 7.42 (d, 1H, *J* = 7.5 Hz, 11-H). ESI-MS *m/z* 364 (M+H)^+^. Anal. Calcd for C_17_H_17_NO_4_S_2_, %: C, 56.18; H, 4.71; N, 3.85. Found, %: C, 56.28, H, 4.65, N, 3.80.

#### Ethyl rel-[(5aR,11bR)-10-bromo-2-oxo-5a,11b-dihydro-2H,5H-chromeno[4′,3′:4,5]thio–pyrano[2,3-d][1,3]thiazol-3(6H)-yl]acetate (**3b**)

Yield 76%, mp 148–150°C (EtOH). ^1^H NMR (400 MHz, DMSO-*d_6_*): 1.30 (t, 3H, CH_3_), 2.28–2.35 (m, 1H, 5a-H), 3.00–3.08 (m, 1H, 5-H), 3.18 (dd, 1H, *J* = 3.8, 11.6 Hz, 5-H), 3.97 (t, 1H, *J* = 10.6 Hz, 6-H), 3.99 (d, 1H, *J* = 10.6 Hz, 11b-H), 4.21 (q, 2H, OCH_2_), 4.40 (d, 2H, CH_2_CO), 4.44 (dd, 1H, *J* = 4.2, 10.6 Hz, 6-H), 6.81 (d, 1H, *J* = 8.0 Hz, 8-H), 7.13 (d, 1H, *J* = 8.0 Hz, 9-H), 7.43 (s, 1H, 11-H). ESI-MS *m/z* 442 and 444 (M+H)^+^. Anal. Calcd for C_17_H_16_BrNO_4_S_2_, %: C, 46.16, H, 3.65, N, 3.17. Found, %: C, 46.28, H, 3.82, N, 3.01.

#### rel-2-[(5aR,11bR)-2-Oxo-5a,11b-dihydro-2H,5H-chromeno[4′,3′:4,5]thiopyrano[2,3-d]–[1,3]thiazol-3(6H)-yl]acetamide (**3e**)

Yield 94%, mp 210–211°C (DMFA-EtOH). ^1^H NMR (400 MHz, DMSO-*d_6_*): 2.21–2.23 (m, 1H, 5a-H), 3.02 (t, 1H, *J* = 11.5 Hz, 5-H), 3.23 (dd, 1H, *J* = 3.7, 11.5 Hz, 5-H), 3.92 (t, 1H, *J* = 10.7 Hz, 6-H), 4.02 (d, 1H, *J* = 10.5 Hz, 11b-H), 4.24 (d, 1H, *J* = 18.2 Hz, CH_2_), 4.28 (d, 1H, *J* = 18.2 Hz, CH_2_), 4.43 (dd, 1H, *J* = 3.7, 10.7 Hz, 6-H), 6.89 (d, 1H, *J* = 7.3 Hz, 8-H), 7.00 (t, 1H, *J* = 7.3 Hz, 10-H), 7.22 (t, 1H, *J* = 7.7 Hz, 9-H), 7.45 (d, 1H, *J* = 7.7 Hz, 11-H), 7.30 (s, 1H, NH), 7.67 (s, 1H, NH). ESI-MS *m/z* 335 (M+H)^+^. Anal. Calcd for C_15_H_14_N_2_O_3_S_2_, %: C, 53.87; H, 4.22; N, 8.38. Found, %: C, 53.72; H, 4.40; N, 8.27.

#### rel-2-[(5aR,11bR)-2-Oxo-5a,11b-dihydro-2H,5H-chromeno[4′,3′:4,5]thiopyrano[2,3-d]–[1,3]thiazol-3(6H)-yl]-N-[3-(trifluoromethyl)phenyl]acetamide (**3f**)

Yield 47%, mp 240–242°C (DMFA-EtOH). ^1^H NMR (400 MHz, DMSO-*d_6_*): 2.22–2.40 (m, 1H, 5a-H), 3.00–3.08 (m, 1H, 5-H), 3.20 (dd, 1H, *J* = 3.8, 12.2 Hz, 5-H), 3.80–3.98 (m, 1H, 6-H), 4.00 (d, 1H, *J* = 10.4 Hz, 11b-H), 4.44 (dd, 1H, *J* = 3.6, 10.3 Hz, 6-H), 4.53 (d, 1H, *J* = 17.0 Hz, CH_2_), 4.59 (d, 1H, *J* = 17.0 Hz, CH_2_), 6.86 (d, 1H, *J* = 7.8 Hz, 8-H), 7.00 (t, 1H, *J* = 7.8 Hz, 10-H), 7.21 (t, 1H, *J* = 7.6 Hz, 9-H), 7.45 (d, 1H, *J* = 7.6 Hz, 11-H), 7.46 (d, 1H, *J* = 7.8 Hz, arom.),7.49 (t, 1H, *J* = 7.8 H, arom.), 7.73 (d, 1H, *J* = 7.8 Hz, arom.), 8.09c (s, 1H, arom.), 11.78 (s, 1H, NH). ^13^C NMR (100 MHz, DMSO-*d*_6_): 28.5, 38.0, 38.9, 46.2, 68.7, 105.4, 115.7, 117.7, 120.5, 121.0, 121.2, 123.9 (d, *J*_CF_ = 242 Hz, 1C), 123.2, 127.6, 128.9, 130.2 (d, *J*_CF_ = 17 Hz, 1C), 130.7, 139.7, 154.9, 165.5, 169.8. ESI-MS *m/z* 479 (M+H)^+^. Anal. Calcd for C_22_H_17_F_3_N_2_O_3_S_2_, %: C, 55.22; H, 3.58; N, 5.85. Found, %: C, 55.39; H, 3.41; N, 6.01.

#### rel-2-[(5aR,11bR)-2-Oxo-5a,11b-dihydro-2H,5H-chromeno[4′,3′:4,5]thiopyrano[2,3-d]–[1,3]thiazol-3(6H)-yl]-N-[2-(trifluoromethyl)phenyl]acetamide (**3g**)

Yield 58%, mp 218–220°C (DMFA-EtOH). ^1^H NMR (400 MHz, DMSO-*d_6_*): 2.19–2.23 (m, 1H, 5a-H), 3.05 (t, 1H, *J* = 11,7 Hz, 5-H), 3.24 (dd, 1H, *J* = 3.5, 11.7 Hz, 5-H), 3,91 (t, 1H, *J* = 10,9 Hz, 6-H), 4.02 (d, 1H, *J* = 10.6 Hz, 11b-H), 4.40 (dd, 1H, *J* = 3.7, 10.9 Hz, 6-H), 4.53 (d, 1H, *J* = 18.4 Hz, CH_2_), 4.58 (d, 1H, *J* = 18.4 Hz, CH_2_),6.88 (d, 1H, *J* = 7.6 Hz, 8-H), 6.98 (t, 1H, *J* = 7.6 Hz, 10-H), 7.18–7.24 (m, 2H, 9-H, 11-H), 7.44–7.52 (m, 2H, arom.), 7.70 (t, 1H, *J* = 7.8 Hz, arom.), 7.76 (d, 1H, *J* = 7.8 Hz, arom.), 10.04 (s, 1H, NH). ESI-MS *m/z* 479 (M+H)^+^. Anal. Calcd for C_22_H_17_F_3_N_2_O_3_S_2,_ %: C, 55.22; H, 3.58; N, 5.85. Found, %: C, 55.09; H, 3.69; N, 5.95.

#### Ethyl rel-4-({[(5aR,11bR)-2-oxo-5a,11b-dihydro-2H,5H-chromeno[4′,3′:4,5]thiopyrano– [2,3-d][1,3]thiazol-3(6H)-yl]acetyl}amino)benzoate (**3h**)

Yield 57%, mp 246–248°C (DMFA-EtOH). ^1^H NMR (400 MHz, DMSO-*d_6_*): 1.34 (t, 3H, CH_3_), 2.20–2.40 (m, 1H, 5a-H), 3.02 (t, 1H, *J* = 10,6 Hz, 5-H), 3.22 (dd, 1H, *J* = 3.9, 12.0 Hz, 5-H), 3.97 (t, 1H, *J* = 11,0 Hz, 6-H), 4.00 (d, 1H, *J* = 10.4 Hz, 11b-H), 4.28 (q, 2H, OCH_2_), 4.42 (dd, 1H, *J* = 3.8, 10.4 Hz, 6-H), 4.47 (d, 1H, *J* = 16.0 Hz, NCH_2_CO), 4.53 (d,1H, *J* = 16.0 Hz, NCH_2_CO), 6.84 (d, 1H, *J* = 8.1 Hz, 8-H), 6.95 (t, 1H, *J* = 8.2 Hz, 10-H), 7.18 (t, 1H, *J* = 7.6 Hz, 9-H), 7.45 (d, 1H, *J* = 7.7 Hz, 11-H), 7.68 (d, 2H, *J* = 8.8 Hz, arom.), 7.90 (d, 2H, *J* = 8.8 Hz, arom.), 10.61 (s, 1H, NH). ^13^C NMR (100 MHz, DMSO-*d*_6_): 14.7, 28.5, 38.0, 38.9, 46.3, 61.0, 68.8, 107.4, 117.7, 119.1, 121.2, 122.9, 124.9, 125.2, 127.6, 128.9, 130.8, 143.2, 154.9, 165.4, 165.7, 169.8. ESI-MS *m/z* 483 (M+H)^+^. Anal. Calcd for C_24_H_22_N_2_O_5_S_2,_ %: C, 59.73; H, 4.60; N, 5.80. Found, %: C, 59.90; H, 4.41; N, 5.96.

#### rel-N-(2-Methoxyphenyl)-2-[(5aR,11bR)-2-oxo-5a,11b-dihydro-2H,5H-chromeno[4′,3′:4,5]–thiopyrano[2,3-d][1,3]thiazol-3(6H)-yl]acetamide (**3i**)

Yield 62%, mp 210–212°C (DMFA:EtOH). ^1^H NMR (400 MHz, DMSO-*d_6_*): 2.20–2.40 (m, 1H, 5a-H), 3.03 (t, 1H, *J* = 10,8 Hz, 5-H), 3.25 (dd, 1H, *J* = 3.8, 12.2 Hz, 5-H), 3.90 (s, 3H, OCH_3_), 3.96 (t, 1H, *J* = 11,0 Hz, 6-H), 4.06 (d, 1H, *J* = 10.3 Hz, 11b-H), 4.42 (dd, 1H, *J* = 3.8, 10.8 Hz, 6-H), 4.52 (d, 1H, *J* = 18,0 Hz, CH_2_), 4.58 (d, 1H, *J* = 18,0 Hz, CH_2_), 6.75-7.06 (m, 3H, arom.), 7.10–7.16 (m, 2H, arom.), 7.22 (d, 1H, *J* = 8.0 Hz, arom.), 7.43 (d, 1H, *J* = 7.8 Hz, 11-H), 8.06 (d, 1H, *J* = 8.0 Hz, arom.), 10.61 (s, 1H, NH). ESI-MS *m/z* 441 (M+H)^+^. Anal. Calcd for C_22_H_20_N_2_O_4_S_2,_ %: C, 59.98; H, 4.58; N, 6.36. Found, %: C, 60.10; H, 4.77; N, 6.45.

#### rel-N-(3-Methoxyphenyl)-2-[(5aR,11bR)-2-oxo-5a,11b-dihydro-2H,5H-chromeno[4′,3′:4,5]–thiopyrano[2,3-d][1,3]thiazol-3(6H)-yl]acetamide (**3j**)

Yield 72%, mp 235–237°C (DMFA-EtOH). ^1^H NMR (400 MHz, DMSO-*d_6_*): 2.22–2.38 (m, 1H, 5a-H), 3.00 (t, 1H, *J* = 11,0 Hz, 5-H), 3.15 (dd, 1H, *J* = 3.9, 12.0 Hz, 5-H), 3.72 (s, 3H, OCH_3_), 3.90 (t, 1H, *J* = 11.0 Hz, 6-H), 3.98 (d, 1H, *J* = 10.4 Hz, 11b-H), 4.40 (dd, 1H, *J* = 3.8, 11.0 Hz, 6-H), 4.44 (d, 1H, *J* = 17.7 Hz, CH_2_), 4.50 (d, 1H, *J* = 17.7 Hz, CH_2_), 6.51 (d, 1H, *J* = 8.7 Hz, arom.), 6.82 (d, 1H, *J* = 7.5 Hz, 8-H), 6,94 (t, 1H, *J* = 7,5 Hz, 10-H), 7,03 (d, 1H, *J* = 8.7 Hz, arom.), 7.18 (t, 1H, *J* = 7,5 Hz, 9-H), 7.20 (t, 1H, *J* = 8,7 Hz, arom.), 7.30 (s, 1H, arom.), 7.46 (t, 1H, *J* = 7.5 Hz, 11-H), 9.40 (s, 1H, NH). ESI-MS *m/z* 441 (M+H)^+^. Anal. Calcd for C_22_H_20_N_2_O_4_S_2,_ %: C, 59.98; H, 4.58; N, 6.36. Found, %: C, 59.91; H, 4.69; N, 6.56.

#### rel-N-(4-Methoxyphenyl)-2-[(5aR,11bR)-2-oxo-5a,11b-dihydro-2H,5H-chromeno[4′,3′:4,5]–thiopyrano[2,3-d][1,3]thiazol-3(6H)-yl]acetamide (**3k**)

Yield 57%, mp 262–264°C (DMFA-EtOH). ^1^H NMR (400 MHz, DMSO-*d_6_*): 2.25–2.38 (m, 1H, 5a-H), 3.00–3.08 (m, 1H, 5-H), 3.15 (dd, 1H, *J* = 4.0, 12.0 Hz, 5-H), 3.73 (s, 3H, OCH_3_), 3.86–3.98 (m, 2H, 6-H and 11b-H), 4.40 (dd, 1H, *J* = 3.8, 11.4 Hz, 6-H), 4.44 (d, 1H, *J* = 17,3 Hz, CH_2_), 4.48 (d, 1H, *J* = 17,3 Hz, CH_2_), 6.76–6.82 (m, 3H, arom.), 6.92 (t, 1H, *J* = 7.7 Hz, 10-H), 7.39 (t, 1H, *J* = 7.7 Hz, 9-H), 7.42–7.48 (m, 3H, arom.), 10.30 (s, 1H, NH). ESI-MS *m/z* 441 (M+H)^+^. Anal. Calcd for C_22_H_20_N_2_O_4_S_2_: % C, 59.98; H, 4.58; N, 6.36. Found, %: C, 59.90; H, 4.70; N, 6.56.

#### rel-N-(2-Methylphenyl)-2-[(5aR,11bR)-2-oxo-5a,11b-dihydro-2H,5H-chromeno[4′,3′:4,5]–thiopyrano[2,3-d][1,3]thiazol-3(6H)-yl]acetamide (**3l**)

Yield 59%, mp 210–212°C (DMFA-EtOH). ^1^H NMR (400 MHz, DMSO-*d_6_*): 2.25 (s, 3H, CH_3_), 2.26–2.40 (m, 1H, 5a-H), 3.00 (t, 1H, *J* = 12,0 Hz, 5-H), 3.23 (dd, 1H, *J* = 3.9, 12.0 Hz, 5-H), 3.94 (t, 1H, *J* = 10,6 Hz, 6-H), 4.06 (d, 1H, *J* = 10.4 Hz, 11b-H), 4.44 (dd, 1H, *J* = 3.8, 11.0 Hz, 6-H), 4.48 (d, 1H, *J* = 18,0 Hz, CH_2_), 4.52 (d, 1H, *J* = 18.0 Hz, CH_2_), 6.73–6.94 (m, 2H, arom.), 7.00–7.22 (m, 4H, arom.), 7.41–7.48 (m, 2H, arom.), 9.98 (s, 1H, NH). ESI-MS *m/z* 425 (M+H)^+^. Anal. Calcd for C_22_H_20_N_2_O_3_S_2,_ %: C, 62.24; H, 4.75; N, 6.60. Found, %: C, 62.44; H, 4.62; N, 6.82.

#### rel-N-(3-Methylphenyl)-2-[(5aR,11bR)-2-oxo-5a,11b-dihydro-2H,5H-chromeno[4′,3′:4,5]–thiopyrano[2,3-d][1,3]thiazol-3(6H)-yl]acetamide (**3m**)

Yield 67%, mp 234–236°C (DMFA-EtOH). ^1^H NMR (400 MHz, DMSO-*d_6_*): 2.25–2.38 (m, 1H, 5a-H), 2.30 (s, 3H, CH_3_), 3.02 (t, 1H, *J* = 11,0 Hz, 5-H), 3.25 (dd, 1H, *J* = 4.0, 12.0 Hz, 5-H), 3.94 (t, 1H, *J* = 10,6 Hz, 6-H), 4.02 (d, 1H, *J* = 10.6 Hz, 11b-H), 4.42 (dd, 1H, *J* = 4.0, 11.0 Hz, 6-H), 4.44 (d, 1H, *J* = 16.9 Hz, CH_2_), 4.48 (d, 1H, *J* = 16.9 Hz, CH_2_), 6.78-6.85 (m, 2H, arom.), 6.93 (t, 1H, *J* = 7.8 Hz, 10-H), 7.08–7.16 (m, 2H, arom.), 7.33 (d, 1H, *J* = 8.8 Hz, arom.), 7.42 (s, 1H, arom.), 7.44 (d, 1H, *J* = 7.8 Hz, 11-H), 10.05 (s, 1H, NH). ^13^C NMR (100 MHz, DMSO-*d*_6_): 21.7, 28.5, 38.0, 38.9, 46.2, 68.8, 105.2, 116.8, 117.7, 120.1, 121.2, 122.9, 124.7, 124.9, 127.6, 128.9, 129.1, 138.5, 139.0, 154.9, 164.7, 169.7. ESI-MS *m/z* 425 (M+H)^+^. Anal. Calcd for C_22_H_20_N_2_O_3_S_2_ %: C, 62.24; H, 4.75; N, 6.60. Found, %: C, 62.32; H, 4.80; N, 6.56.

#### rel-N-(4-Methylphenyl)-2-[(5aR,11bR)-2-oxo-5a,11b-dihydro-2H,5H-chromeno[4′,3′:4,5]–thiopyrano[2,3-d][1,3]thiazol-3(6H)-yl]acetamide (**3n**)

Yield 68%, mp 230–233°C (DMFA-EtOH). ^1^H NMR (400 MHz, DMSO-*d_6_*): 2.27 (s, 3H, CH_3_), 2.65–2.85 (m, 1H, 5a-H), 3.00 (t, 1H, *J* = 11,4 Hz, 5-H), 3.24 (dd, 1H, *J* = 3.8, 11.9 Hz, 5-H), 3.89 (t, 1H, *J* = 11.4 Hz, 6-H), 3.98 (d, 1H, *J* = 11.4 Hz, 11b-H), 4.42 (dd, 1H, *J* = 3.9, 11.4 Hz, 6-H), 4.44 (d, 1H, *J* = 18.2 Hz, CH_2_), 4.48 (d, 1H, *J* = 18.2 Hz, CH_2_), 6.83 (d, 1H, *J* = 8.6 Hz, 8-H), 6.95 (t, 1H, *J* = 8,6 Hz, 10-H), 7.08 (d, 2H, *J* = 8.4 Hz, arom.), 7.18 (t, 1H, *J* = 8.6 Hz, 9-H), 7.40–7.50 (m, 3H, arom.), 10.15 (s, 1H, NH). ^13^C NMR (100 MHz, DMSO-*d*_6_): 20.9, 28.5, 38.0, 39.0, 46.1, 68.8, 105.3, 117.7, 119.6, 121.2, 122.9, 125.0, 127.7, 128.9, 129.7, 133.1, 136.5, 154.9, 164.5, 169.7. ESI-MS *m/z* 425 (M+H)^+^. Anal. Calcd for C_22_H_20_N_2_O_3_S_2,_ %: C, 62.24; H, 4.75; N, 6.60. Found, %: C, 62.38; H, 4.55; N, 6.67.

#### rel-N-(4-Fluorophenyl)-2-[(5aR,11bR)-2-oxo-5a,11b-dihydro-2H,5H-chromeno[4′,3′:4,5]–thiopyrano[2,3-d][1,3]thiazol-3(6H)-yl]acetamide (**3o**)

Yield 74%, mp 290–291°C (DMFA-EtOH). ^1^H NMR (400 MHz, DMSO-*d_6_*): 2.20–2.38 (m, 1H, 5a-H), 3.02 (t, 1H, *J* = 11.5 Hz, 5-H), 3.25 (dd, 1H, *J* = 3.8, 11.8 Hz, 5-H), 3.89 (t, 1H, *J* = 11.2 Hz, 6-H), 3.98 (d, 1H, *J* = 10.8 Hz, 11b-H), 4.40 (dd, 1H, *J* = 3.9, 11.4 Hz, 6-H), 4.44 (d, 1H, *J* = 18.2 Hz, CH_2_), 4.50 (d, 1H, *J* = 18.2 Hz, CH_2_), 6.84 (d, 1H, *J* = 8.1 Hz, 8-H), 6.95 (t, 1H, *J* = 8.0 Hz, 10-H), 7.17 (t, 1H, *J* = 8.1 Hz, 9-H), 7.49 (d, 1H, *J* = 7.8 Hz, 11-H), 7.07 (t, 2H, *J* = 8.4 Hz, arom.), 7.57 (dd, 2H, J = 5.1, 9.0 Hz, arom.),10.31 (s, 1H, NH). ^13^C NMR (100 MHz, DMSO-*d*_6_): 28.5, 38.0, 38.9, 46.1, 68.8, 105.3, 115,9 (d, *J*_CF_ = 22.4 Hz, 2C), 117.7, 121.2, 121.4 (d, *J*_CF_ = 7.8 Hz, 2C), 122.9, 124.9, 127.7, 128.9, 135.4, 154.9, 158.7 (d, *J*_CF_ = 240 Hz, 1C), 164.7, 169.7. ESI-MS *m/z* 429 (M+H)^+^. Anal. Calcd for C_21_H_17_FN_2_O_3_S_2,_ %: C, 58.86; H, 4.00; N, 6.54. Found, %: C, 58.90; H, 3.87; N, 6.43.

#### rel-N-(4-Chlorophenyl)-2-[(5aR,11bR)-2-oxo-5a,11b-dihydro-2H,5H-chromeno[4′,3′:4,5]–thiopyrano[2,3-d][1,3]thiazol-3(6H)-yl]acetamide (**3p**)

Yield 50%, mp 260–263°C (DMFA-EtOH). ^1^H NMR (400 MHz, DMSO-*d_6_*): 2.25–2.42 (m, 1H, 5a-H), 2.61 (t, 1H, *J* = 11.0 Hz, 5-H), 3.25 (dd, 1H, *J* = 4.0, 12.0 Hz, 5-H), 3.82 (t, 1H, *J* = 10.6 Hz, 6-H), 4.02 (d, 1H, *J* = 10.6 Hz, 11b-H), 4.22 (dd, 1H, *J* = 4.0, 11.0 Hz, 6-H), 4.46 (d, 1H, *J* = 18.0 Hz, CH_2_), 4.52 (d, 1H, *J* = 18.0 Hz, CH_2_), 6.79 (d, 1H, *J* = 8.2 Hz, 8-H), 6.90 (t, 1H, *J* = 7.4 Hz, 10-H), 7.16 (t, 1H, *J* = 7.8 Hz, 9-H), 7.18 (d, 1H, *J* = 7.6 Hz, 11-H), 7.29 (d, 2H, J = 7.4 Hz, arom.), 7.54 (d, 2H, J = 7.4 Hz, arom.), 10.15 (s, 1H, NH). ^13^C NMR (100 MHz, DMSO-*d*_6_): 28.5, 38.0, 38.9, 46.2, 68.8, 105.3, 117.3, 117.7, 121.2, 122.9, 124.9, 127.7, 128.9, 129.2, 130.3, 138.0, 154.9, 165.0, 169.7. ESI-MS *m/z* 445 and 447 (M+H)^+^. Anal. Calcd for C_21_H_17_ClN_2_O_3_S_2,_ %: C, 56.69; H, 3.85; N, 6.30. Found, %: C, 56.80, H, 3.95, N, 6.21.

#### rel-N-(3,4-Dichlorophenyl)-2-[(5aR,11bR)-2-oxo-5a,11b-dihydro-2H,5H-chromeno–[4′,3′:4,5]thiopyrano[2,3-d][1,3]thiazol-3(6H)-yl]acetamide (**3q**)

Yield 47%, mp 238–240°C (DMFA-EtOH). ^1^H NMR (400 MHz, DMSO-*d_6_*): 2.25–2.38 (m, 1H, 5a-H), 3.02 (t, 1H, *J* = 11.0 Hz, 5-H), 3.25 (dd, 1H, *J* = 3.8, 11.0 Hz, 5-H), 3.95 (t, 1H, *J* = 11.4 Hz, 6-H), 4.10 (d, 1H, *J* = 11.0 Hz, 11b-H), 4.40 (dd, 1H, *J* = 3.9, 11.4 Hz, 6-H), 4.44 (d, 1H, *J* = 18.4 Hz, CH_2_), 4.50 (d, 1H, *J* = 18.4 Hz, CH_2_), 6.86 (d, 1H, *J* = 8.0 Hz, 8-H), 7.00 (t, 1H, *J* = 8.0 Hz, 10-H), 7.20 (t, 1H, *J* = 8.1 Hz, 9-H), 7.50 (d, 1H, *J* = 7.8 Hz, 11-H), 7.56–7.62 (m, 2H, arom.), 8.02 (s, 1H, arom.), 10.05 (s, 1H, NH). ESI-MS *m/z* 479 and 481 (M+H)^+^. Anal. Calcd for C_21_H_16_Cl_2_N_2_O_3_S_2,_ %: C, 52.61; H, 3.36; N, 5.84. Found, %: C, 52.80; H, 3.20; N, 5.88.

#### rel-N-(2,4-Dichlorophenyl)-2-[(5aR,11bR)-2-oxo-5a,11b-dihydro-2H,5H-chromeno–[4′,3′:4,5]thiopyrano[2,3-d][1,3]thiazol-3(6H)-yl]acetamide (**3r**)

Yield 52%, mp 265–267°C (DMFA:EtOH). ^1^H NMR (400 MHz, DMSO-*d_6_*): 2.23–2.34 (m, 1H, 5a-H), 2.98 (t, 1H, *J* = 11.0 Hz, 5-H), 3.24 (dd, 1H, *J* = 3.8, 11.0 Hz, 5-H), 4.05 (t, 1H, *J* = 11.4 Hz, 6-H), 4.15 (d, 1H, *J* = 10.6 Hz, 11b-H), 4.42 (dd, 1H, *J* = 3.9, 11.4 Hz, 6-H), 4.44 (d, 1H, *J* = 17.6 Hz, CH_2_), 4.50 (d, 1H, *J* = 17.6 Hz, CH_2_), 6.89 (d, 1H, *J* = 7.8 Hz, 8-H), 7.05 (t, 1H, *J* = 7.8 Hz, 10-H), 7.22 (t, 1H, *J* = 7.8 Hz, 9-H), 7.45 (d, 1H, *J* = 8.0 Hz, arom.), 7.48 (d, 1H, *J* = 7.8 Hz, 11-H), 7.97 (s, 1H, arom.), 8.22 (d, 1H, *J* = 8.0 Hz, arom.), 10.05 (s, 1H, NH). ESI-MS *m/z* 479 and 481 (M+H)^+^. Anal. Calcd for C_21_H_16_Cl_2_N_2_O_3_S_2,_ %: C, 52.61; H, 3.36; N, 5.84. Found, %: C, 52.74; H, 3.47; N, 5.86.

#### rel-N-(4-Acetylphenyl)-2-[(5aR,11bR)-10-bromo-2-oxo-5a,11b-dihydro-2H,5H-chromeno–[4′,3′:4,5]thiopyrano[2,3-d][1,3]thiazol-3(6H)-yl]acetamide (**3s**)

Yield 65%, mp 230–232°C (DMFA-EtOH). ^1^H NMR (400 MHz, DMSO-*d_6_*): 2.51 (s, 3H, CH_3_CO), 2.25–2.35 (m, 1H, 5a-H), 3.00–3.08 (m, 1H, 5-H), 3.16 (dd, 1H, *J* = 3.8, 11.6 Hz, 5-H), 3.93 (t, 1H, *J* = 11.0 Hz, 6-H), 3.98 (d, 1H, *J* = 11.0 Hz, 11b-H), 4.43 (dd, 1H, *J* = 3.8, 11.0 Hz, 6-H), 4.48 (d, 1H, *J* = 18.6 Hz, CH_2_), 4.52 (d, 1H, *J* = 18.6 Hz, CH_2_), 6.80 (d, 1H, *J* = 8.0 Hz, 8-H), 7.25 (d, 1H, *J* = 8.0 Hz, 9-H), 7.43 (s, 1H, 11-H), 7.68 (d, 2H, *J* = 8.0 Hz, arom.), 7.88 (d, 2H, *J* = 8.0 Hz, arom.), 10.51 (s, 1H, NH). ^13^C NMR (100 MHz, DMSO-*d*_6_): 26.9, 28.3, 37.9, 38.8, 46.4, 69.2, 104.3, 118.9, 119.5, 124.6, 125.3, 125.4, 126.9, 128.7, 130.1, 132.6, 143.2, 153.8, 165.4, 169.5, 196.9. ESI-MS *m/z* 531 and 533 (M+H)^+^. Anal. Calcd for C_23_H_20_Cl_2_N_2_O_4_S_2_, %: C, 51.98; H, 3.60; N, 5.27. Found, %: C, 52.10; H, 3.61; N, 5.35.

#### rel-N-(4-Methylphenyl)-2-[(5aR,11bR)-10-nitro-2-oxo-5a,11b-dihydro-2H,5H-chromeno–[4′,3′:4,5]thiopyrano[2,3-d][1,3]thiazol-3(6H)-yl]acetamide (**3t**)

Yield 45%, mp 248–250°C (DMFA-EtOH). ^1^H NMR (400 MHz, DMSO-*d_6_*): 2.51 (s, 3H, CH_3_CO), 2.40–2.44 (m, 1H, 5a-H), 3.06 (t, 1H, *J* = 11.2 Hz, 5-H), 3.25 (d, 1H, *J =* 11.3 Hz, 5-H), 4.05–4.20 (m, 2H, 6-H), 4.49 (s, 2H, CH_2_), 4.59 (d, 1H, *J* = 11.0 Hz, 11b-H), 7.08 (d, 1H, *J* = 8.0 Hz, 8-H), 7.10 (d, 2H, *J* = 7.8 Hz, arom.), 7.43 (d, 2H, *J* = 7.8 Hz, arom.), 8.10 (d, 1H, *J* = 8.0 Hz, 9-H), 8.43 (s, 1H, 11-H), 10.23 (s, 1H, NH). ^13^C NMR (100 MHz, DMSO-*d*_6_): 20.9, 27.9, 37.8, 38.5, 46.2, 70.5, 102.9, 118.4, 119.6, 122.9, 124.7, 124.9, 126.2, 129.7, 133.1, 136.8, 140.8, 160.6, 164.4, 169.5. ESI-MS *m/z* 470 (M+H)^+^. Anal. Calcd for C_22_H_19_N_3_O_5_S_2_, %: C, 56.28; H, 4.08; N, 8.95. Found, %: C, 56.40; H, 4.23; N, 8.89.

### Preparation of compound 4

To 0,01 mol of **1a** the mixture of 50 ml of pyridine and 10 ml of water containing 3 ml of acrylonitrile was added. Reaction mixture was refluxed for 6 h and then precipitated with water. The deposited solid was collected and crystallized from ethanol.

#### rel-3-[(5aR,11bR)-2-Oxo-5a,11b-dihydro-2H,5H-chromeno[4′,3′:4,5]thiopyrano[2,3-d]–[1,3]thiazol-3(6H)-yl]propanenitrile (**4**)

Yield 72%, mp 115–120°C (EtOH). ^1^H NMR (400 MHz, DMSO-*d_6_*): 2.18–2.30 (m, 1H, 5a-H), 2.88 (t, 2H, CH_2_CH_2_CN), 3.05 (t, 1H, *J* = 10.6 Hz, 5-H), 3.30 (dd, 1H, *J* = 3.8, 10.6 Hz, 5-H), 3.90 (t, 1H, *J* = 10.4 Hz, 6-H), 3.92 (t, 2H, CH_2_CH_2_CN), 4.10 (d, 1H, *J* = 10.4 Hz, 11b-H), 4.42 (dd, 1H, *J* = 3.7, 10.4 Hz, 6-H), 6.82 (d, 1H, *J* = 8.0 Hz, 8-H), 6.94 (t, 1H, *J* = 8.0 Hz, 10-H), 7.17 (t, 1H, *J* = 7.6 Hz, 9-H), 7.40 (d, 1H, *J* = 7.6 Hz, 11-H). ^13^C NMR (100 MHz, DMSO-*d*_6_): 17.3, 28.6, 38.1, 38.7, 39.5, 68.7, 106.5, 117.7, 118.4, 121.2, 122.8, 124.1, 127.7, 128.9, 154.9, 169.4. ESI-MS *m/z* 331 (M+H)^+^. Anal. Calcd for C_16_H_14_N_2_O_2_S_2_, %: C, 58.16; H, 4.27; N, 8,48. Found, %: C, 58.20; H, 4.32; N, 8,43.

### Preparation of compound 5

The solution of **4** (0.01 mol) in 30 ml of acetic acid and 15 ml of hydrochloric acid was refluxed for 3 h. After cooling the reaction mixture was precipitated with water. After 24 h, the white solid was collected by filtration and treated with toluene, whereupon it was crystallized from ethanol.

#### rel-3-[(5aR,11bR)-2-Oxo-5a,11b-dihydro-2H,5H-chromeno[4′,3′:4,5]thiopyrano[2,3-d]–[1,3]thiazol-3(6H)-yl]propanoic acid (**5**)

Yield 75%, mp 107–109°C (EtOH). ^1^H NMR (400 MHz, DMSO-*d_6_*): 2.60–2.80 (m, 1H, 5a-H), 3.05 (t, 1H, *J* = 10.8 Hz, 5-H), 3.30 (dd, 1H, *J* = 3.6, 10.8 Hz, 5-H), 3.40 (t, 2H, CH_2_CH_2_), 3.70 (t, 2H, CH_2_CH_2_), 4.05 (d, 1H, *J* = 10.8 Hz, 11b-H), 4.15 (t, 1H, *J* = 10.4 Hz, 6-H), 4.30 (dd, 1H, *J* = 3.7, 10.4 Hz, 6-H), 6.78 (d, 1H, *J* = 8.0 Hz, 8-H), 6.90 (t, 1H, *J* = 8.0 Hz, 10-H), 7.15 (t, 1H, *J* = 7.6 Hz, 9-H), 7.20 (d, 1H, *J* = 7.6 Hz, 11-H), 12.35 (s, 1H, COOH). Anal. Calcd for C_16_H_15_NO_4_S_2_, %: C, 55.0; H, 4.33; N, 4.01. ESI-MS *m/z* 348 (M–H)^−^. Found, %: C, 54.97; H, 4.28; N, 4.00.

### Preparation of Compounds 6a–c

To the solution of **5** (0.01 mol) in anhydrous dioxane thionyl chloride (0.057 mol) was added. Reaction mixture was refluxed for 30 min, after cooling it was precipitated with hexane. Obtained acid chloride was used for further transformations without additional purification. To the solution of acid chloride (0.01 mol) in 10 ml of dioxane a mixture of appropriate aniline (0.01 mol) and triethylamine in 10 ml of dioxane was added. Reaction mixture was heated for 10 min at 100°C and poured into water. Filtered precipitate was crystallized from acetic acid.

#### rel-N-(4-Chlorophenyl)-3-[(5aR,11bR)-2-oxo-5a,11b-dihydro-2H,5H-chromeno[4′,3′:4,5]–thiopyrano[2,3-d][1,3]thiazol-3(6H)-yl]propanamide (**6a**)

Yield 55%, mp 230–232°C (EtOH). ^1^H NMR (400 MHz, DMSO-*d_6_*): 2.60–2.80 (m, 1H, 5a-H), 2.61 (t, 2H, CH_2_CH_2_), 3.23 (t, 1H, *J* = 12.8 Hz, 5-H), 3.43 (dd, 1H, *J* = 3.4, 12.8 Hz, 5-H), 3.82 (t, 2H, CH_2_CH_2_), 4.12 (d, 1H, *J* = 10.5 Hz, 11b-H), 4.02 (t, 1H, *J* = 10.5 Hz, 6-H), 4.23 (dd, 1H, *J* = 3.1, 10.5 Hz, 6-H), 6.79 (d, 1H, *J* = 8.1 Hz, 8-H), 6.90 (t, 1H, *J* = 7.4 Hz, 10-H), 7.15 (t, 1H, *J* = 7.4 Hz, 9-H), 7.18 (d, 1H, *J* = 8.1 Hz, 11-H), 7.32 (d, 2H, *J* = 7.6 Hz, arom.), 7.54 (d, 2H, *J* = 7.6 Hz, arom.), 10.05 (s, 1H, NH). ^13^C NMR (100 MHz, DMSO-*d*_6_): 27.8, 30.0, 33.9, 35.8, 66.2, 105.1, 117.3, 121.0, 121.1, 121.3, 123.2, 127.3, 129.0, 129.1, 130.3, 138.3, 153.6, 168.6, 169.6. ESI-MS *m/z* 459 and 461 (M+H)^+^. Anal. Calcd for C_22_H_19_ClN_2_O_3_S_2_, %: C, 57.57; H, 4.17; N, 6.10. Found, %: C, 57.60; H, 4.13; N, 6.20.

#### rel-N-(4-Hydroxyphenyl)-3-[(5aR,11bR)-2-oxo-5a,11b-dihydro-2H,5H-chromeno[4′,3′:4,5]–thiopyrano[2,3-d][1,3]thiazol-3(6H)-yl]propanamide (**6b**)

Yield 65%, mp 216–218°C (EtOH). ^1^H NMR (400 MHz, DMSO-*d_6_*): 2.60–2.80 (m, 1H, 5a-H), 2.63 (t, 2H, CH_2_CH_2_), 3.09 (t, 1H, *J* = 12.8 Hz, 5-H), 3.30 (dd, 1H, *J* = 3.4, 12.8 Hz, 5-H), 3.80 (t, 2H, CH_2_CH_2_), 4.15 (d, 1H, *J* = 10.5 Hz, 11b-H), 4.05 (t, 1H, *J* = 10.5 Hz, 6-H), 4.30 (dd, 1H, *J* = 3.1, 10.5 Hz, 6-H), 6.78 (d, 1H, *J* = 8.0 Hz, 8-H), 6.90 (t, 1H, *J* = 7.4 Hz, 10-H), 7.15 (t, 1H, *J* = 7.4 Hz, 9-H), 7.20 (d, 1H, *J* = 8.0 Hz, 11-H), 7.36 (d, 2H, arom.), 7.55 (d, 2H, arom.), 8.43 (s, 1H, OH), 10.05 (s, 1H, NH). ^13^C NMR (100 MHz, DMSO-*d*_6_): 27.8, 30.0, 33.9, 35.6, 66.2, 105.1, 115.5, 117.3, 121.0, 121.2, 121.6, 123.2, 129.1, 130.3, 131.0, 153.6, 153.9, 167.6, 169.6. ESI-MS *m/z* 441 (M+H)^+^. Anal. Calcd for C_22_H_19_ClN_2_O_3_S_2_, %: C, 59.98; H, 4.58; N, 6.36. Found, %: C, 59.88; H, 4.56; N, 6.48.

#### rel-3-[(5aR,11bR)-2-Oxo-5a,11b-dihydro-2H,5H-chromeno[4′,3′:4,5]thiopyrano[2,3-d]–[1,3]thiazol-3(6H)-yl]-N-(4-sulfamoylphenyl)propanamide (**6c**)

Yield 65%, mp 224–226°C (EtOH). ^1^H NMR (400 MHz, DMSO-*d_6_*): 2.60–2.80 (m, 1H, 5a-H), 2.60 (t, 2H, CH_2_CH_2_), 3.20 (t, 1H, *J* = 12.8 Hz, 5-H), 3.30 (dd, 1H, *J* = 3.4, 12.8 Hz, 5-H), 3.78 (t, 2H, CH_2_CH_2_), 4.10 (d, 1H, *J* = 10.5 Hz, 11b-H), 4.04 (t, 1H, *J* = 10.5 Hz, 6-H), 4.32 (dd, 1H, *J* = 3.1, 10.5 Hz, 6-H), 6.78 (d, 1H, *J* = 8.1 Hz, 8-H), 6.92 (t, 1H, *J* = 7.6 Hz, 10-H), 7.14 (t, 1H, *J* = 7.6 Hz, 9-H), 7.18 (d, 1H, *J* = 8.1 Hz, 11-H), 7.34 (d, 2H, *J* = 7.6 Hz, arom.), 7.52 (d, 2H, *J* = 7.6 Hz, arom.), 7.54 (s, 2H, SO_2_NH_2_), 10.05 (s, 1H, NH). ESI-MS *m/z* 505 (M+H)^+^. Anal. Calcd for C_22_H_19_ClN_2_O_3_S_2_, %: C, 52.47; H, 4.20; N, 8.34. Found, %: C, 52.58; H, 4.11; N, 8.30.

### Cytotoxic activity against malignant human tumor cells

Primary anticancer *in vitro* assay was performed at human tumor cell lines panel derived from nine neoplastic diseases, in accordance with the protocol of the Drug Evaluation Branch, National Cancer Institute, Bethesda [15–18]. Tested compounds were added to the culture at a single concentration (10^−5^ M) and the cultures were incubated for 48 h. End point determinations were made with a protein binding dye, sulforhodamine B (SRB). Results for each tested compound were reported as the percent of growth of the treated cells when compared to the untreated control cells. The percentage growth was evaluated spectrophotometrically versus controls not treated with test agents.

The cytotoxic and/or growth inhibitory effects of the most active selected compounds were tested in vitro against the full panel of about 60 human tumor cell lines at 10-fold dilutions of five concentrations ranging from 10^−4^ to 10^−8^ M. A 48-h continuous drug exposure protocol was followed and an SRB protein assay was used to estimate cell viability or growth. Using the seven absorbance measurements [time zero, (Tz), control growth in the absence of drug, (C), and test growth in the presence of drug at the five concentration levels (Ti)], the percentage growth was calculated at each of the drug concentration levels. Percentage growth inhibition was calculated as: [(Ti − Tz) / (C − Tz)] × 100 for concentrations for which Ti ≥ Tz, [(Ti − Tz) / Tz] × 100 for concentrations for which Ti < Tz. Three dose response parameters were calculated for each compound. Growth inhibition of 50% (GI_50_) was calculated from [(*Ti* − *Tz*)/(*C* − *Tz*)] × 100 = 50, which is the drug concentration resulting in a 50% lower net protein increase in the treated cells (measured by SRB staining) as compared to the net protein increase seen in the control cells. The drug concentration resulting in total growth inhibition (TGI) was calculated from *Ti* = *Tz*. The LC_50_ (concentration of drug resulting in a 50% reduction in the measured protein at the end of the drug treatment as compared to that at the beginning) indicating a net loss of cells following treatment was calculated from [(*Ti* − *Tz*) / *Tz*] × 100 = -50. Values were calculated for each of these three parameters if the level of activity is reached; however, if the effect was not reached or was exceeded, the value for that parameter was expressed as greater or less than the maximum or minimum concentration tested. The logGI_50_, logTGI, logLC_50_ were then determined, defined as the mean of the logs of the individual GI_50_, TGI, LC_50_ values. The lowest values are obtained with the most sensitive cell lines.

## Figures and Tables

**Sch. 1:**
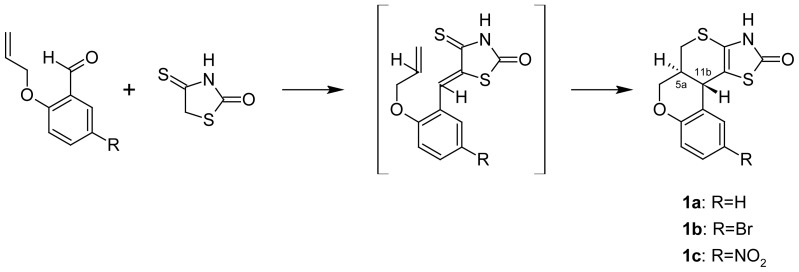
Thiopyrano[2,3-*d*]thiazoles **1a–c** obtained in the Knoevenagel-*hetero*-Diels-Alder reaction.

**Sch. 2:**
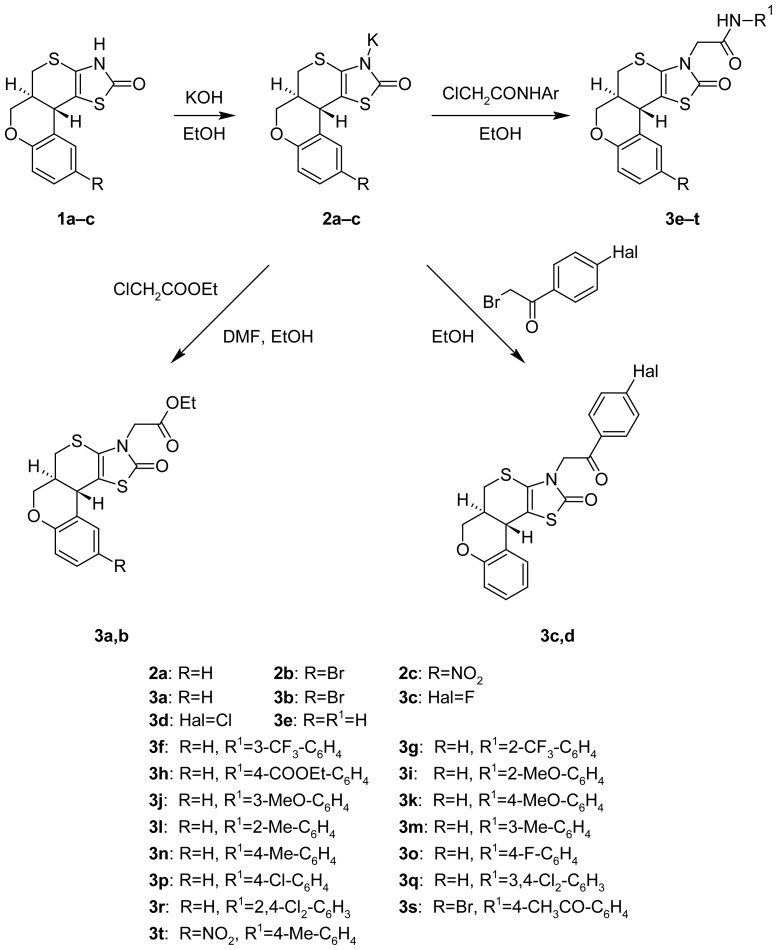
Synthesis of 3-substituted thiopyrano[2,3-*d*]thiazoles.

**Sch. 3:**
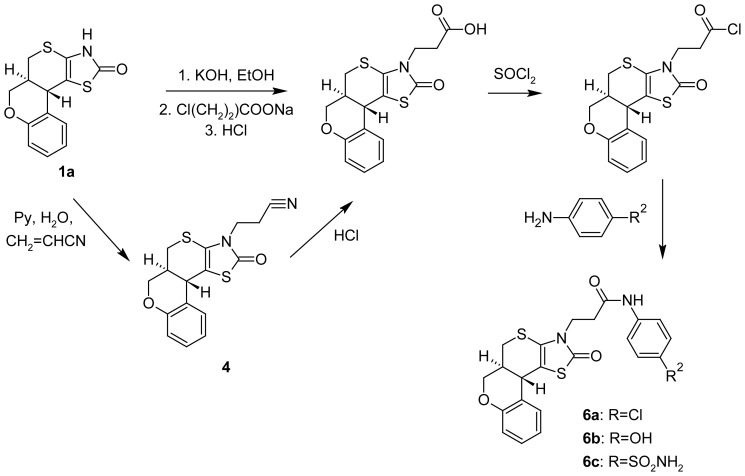
Two alternative methods for the synthesis of **5** and its further modification.

**Tab. 1:** Anticancer activity screening at one dose assay (10^−5^ M)

Comp.	Average growth %	Range of growth, %	Most sensitive cancer cell lines, (growth %)
**3a**	54.59	−16.33–112.90	Ovarian cancer: OVCAR-3 (−16.33)
			Leukemia: SR (−12.33), K-562 (16.19)
			Renal cancer: CAKI-1 (−0.80)
			Breast cancer: MDA-MB-435 (2.23)
			Colon cancer: HT-29 (15.51)

**3b**	49.23	7.34–101.18	Leukemia: CCRF-CEM (7.34), SR (10.12), K-562 (10.55);
			Breast cancer: MDA-MB-435 (7.52)
			Colon cancer: HT-29 (9.91)

**3c**	84.35	39.80–119.29	Breast cancer: T-47D (39.80)

**3d**	97.99	58.48–308.17	Colon cancer: HCT-15 (58.48)
			Melanoma: LOX IMVI (58.59)

**3e**	104.03	72.70–131.64	Leukemia: SR (72.70)

**3j**	99.79	70.81–129.01	Melanoma: UACC-62 (70.81)

**3k**	99.66	72.14–190.72	Ovarian cancer: OVCAR-5 (72.14)

**3l**	102.99	60.33–297.26	Leukemia: SR (60.33)

**4**	101.04	8.59–304.74	Breast cancer: HS 578T (8.59)
			Non-small cell lung cancer: EKVW (39.22)
			CNS cancer: SF-268 (58.00)

**6a**	91.69	60.09–126.78	CNS cancer: SNB-75 (60.09)
			Melanoma: UACC-62 (63.95)
			Renal cancer: UO-31 (68.26)

**6b**	96.02	68.99–143.74	Renal cancer: UO-31 (68.99)

**6c**	103.25	79.97–132.03	Leukemia: SR (79.97)

**Tab. 2:** Total values of the in-depth *in vitro* screening in 5 concenrations (10^−4^–10^−8^ M)

Comp.	NSC NCI code	Average values of the activity parameters			Quantity of the “sensitive” lines (s/t, %)^a^

		logGI_50_	logTGI	logLC_50_	
**3a**	741023	−5.10	−4.14	−4.00	57/57, 100%
**3b**	741958	−4.92	−4.29	−4.01	57/57, 100%
**3f**	735629	−6.22	−4.82	−4.21	57/57, 100%
**3h**	735667	−4.49	−4.09	−4.01	50/56, 89.3%
**3i**	735711	−4.84	−4.11	−4.01	54/57, 94.7%
**3n**	735666	−4.14	−4.12	−4.07	3/53, 5.7%
**3p**	735621	−4.26	−4.03	−4.00	39/58, 67.2%
**3q**	741952	−5.39	−4.45	−4.04	58/58, 100%
**3r**	735709	−4.18	−4.00	−4.00	16/57, 28%

a s/t – ratio of sensitive lines (logGI_50_< −4.00) to the total number of tested lines.

**Tab. 3:** Selectivity ratios for the „hit-compounds” **3a**, **3f** and **3q**

Comp.	Cancer cell lines	GI_50_ (C, μM)	SR^a^	TGI (C, μM)	SR^b^
**3a**	Leukemia	5.4	2.06	80.40	1.05
	Non-small cell lung cancer	10.97	1.02	87.97	0.96
	Colon cancer	6.15	1.81	94.80	0.89
	CNS cancer	6.85	1.63	51.00	1.66
	Melanoma	15.59	0.71	81.40	1.04
	Ovarian cancer	16.81	0.66	92.93	0.91
	Renal cancer	13.68	0.81	93.54	0.90
	Prostate cancer	7.14	1.56	100.00	0.84
	Breast cancer	12.05	0.92	82.70	1.02

**3f**	Leukemia	0.67	1.80	16.83	1.34
	Non-small cell lung cancer	0.55	2.18	21.83	1.04
	Colon cancer	0.65	1.85	16.87	1.34
	CNS cancer	0.52	2.31	23.65	0.96
	Melanoma	0.63	1.90	17.50	1.30
	Ovarian cancer	2.82	0.43	41.75	0.54
	Renal cancer	3.03	0.40	29.29	0.77
	Prostate cancer	0.37	3.24	10.95	2.07
	Breast cancer	0.47	2.55	13.07	1.74

**3q**	Leukemia	3.14	1.61	20.92	2.50
	Non-small cell lung cancer	4.86	1.04	45.68	1.14
	Colon cancer	5.73	0.88	79.68	0.65
	CNS cancer	7.45	0.68	62.10	0.84
	Melanoma	4.96	1.02	58.48	0.89
	Ovarian cancer	5.23	0.97	58.89	0.88
	Renal cancer	6.13	0.83	65.84	0.79
	Prostate cancer	3.05	1.66	11.82	4.41
	Breast cancer	3.32	1.53	26.51	1.97

a Selectivity ratio at the GI_50_ level;

b selectivity ratio at the TGI level.

**Tab. 4:** Results of the COMPARE analysis at the GI_50_ level.

Comp.	PCC	Target	Target vector NSC	N^a^	Target mechanism of action
**3a**	0.515	Macbecin II	S330500	49	Heat shock protein Hsp90 protein inhibition

**3b**	0.571	Macbecin II	S330500	50	Heat shock protein Hsp90 protein inhibition
	0.530	D-Tetrandrine	S77037	52	Inductor of apoptosis, reversal activity for MDR tumors
	0.506	Tamoxifen	S180973	58	Tamoxifen competitively binds to estrogen receptors on tumors and other tissue targets, producing a nuclear complex that decreases DNA synthesis and inhibits estrogen effects.
	0.540	Maytansine	S153858	56	Potent microtubule-targeted compound that induces mitotic arrest

**3f**	0.686	Rhizoxin	S332598	44	Rhizoxin binds beta β-tubulin in eukaryotic cells disrupting microtubule formation.

a number of tested lines.
